# Advancements and Future Prospects in Ocean Wave Energy Harvesting Technology Based on Micro-Energy Technology

**DOI:** 10.3390/mi15101199

**Published:** 2024-09-27

**Authors:** Weihong Yang, Jiaxin Peng, Qiulin Chen, Sicheng Zhao, Ran Zhuo, Yan Luo, Lingxiao Gao

**Affiliations:** 1 China Southern Power Grid Research Institute Co., Ltd., National Engineering Research Center of UHV Technology, New Electrical Equipment Basis of China Southern Power Grid Research Institute Co., Ltd., Guangzhou 510080, China; yangwh@csg.cn (W.Y.); chenql5@csg.cn (Q.C.); zhaosc@csg.cn (S.Z.); zhuoran@csg.cn (R.Z.); luoyan1@csg.cn (Y.L.); 2 School of Mechanical Engineering, Hebei University of Technology, Tianjin 300401, China; 202321203015@stu.hebut.edu.cn

**Keywords:** ocean wave energy harvesting, micro-energy technology, the current research status, the future development trajectory

## Abstract

Marine wave energy exhibits significant potential as a renewable resource due to its substantial energy storage capacity and high energy density. However, conventional wave power generation technologies often suffer from drawbacks such as high maintenance costs, cumbersome structures, and suboptimal conversion efficiencies, thereby limiting their potential. The wave power generation technologies based on micro-energy technology have emerged as promising new approaches in recent years, owing to their inherent advantages of cost-effectiveness, simplistic structure, and ease of manufacturing. This paper provides a comprehensive overview of the current research status in wave energy harvesting through micro-energy technologies, including detailed descriptions of piezoelectric nanogenerators, electromagnetic generators, triboelectric nanogenerators, dielectric elastomer generators, hydrovoltaic generators, and hybrid nanogenerators. Finally, we provide a comprehensive overview of the prevailing issues and challenges associated with these technologies, while also offering insights into the future development trajectory of wave energy harvesting technology.

## 1. Introduction

Energy is a pivotal resource in the contemporary world, and its demand has witnessed significant escalation due to rapid societal advancements [1]. The active exploration of green and clean energy, along with the development of sustainable energy collection systems, are imperative to address the dual challenges posed by environmental pollution and energy shortage [2]. The development of clean energy will mitigate carbon dioxide emissions, effectively alleviate the global energy demand crisis, and reshape the global energy landscape [3]. The renewable nature of marine energy renders it less detrimental to the environment compared to conventional energy sources, thereby garnering extensive attention [4,5,6]. Wave energy, as a distinct form of oceanic energy, represents a pivotal and sustainable source within the realm of oceanic resources. The dynamic motion generated by turbulent ocean waves yields vast, perpetual, and environmentally benign energy potential [7,8]. If harnessed to its full extent alongside other surface wave energies, the future prospects for global energy resources would be immense and promising. However, the intricate nature of the marine environment and the substantial spatiotemporal variability in wave properties (including wave height, period, and direction) pose significant challenges to the acquisition of wave energy. Through collaborative efforts among researchers worldwide, diverse forms of devices for generating wave energy have been developed [9,10,11,12,13,14].

**Figure 1:**
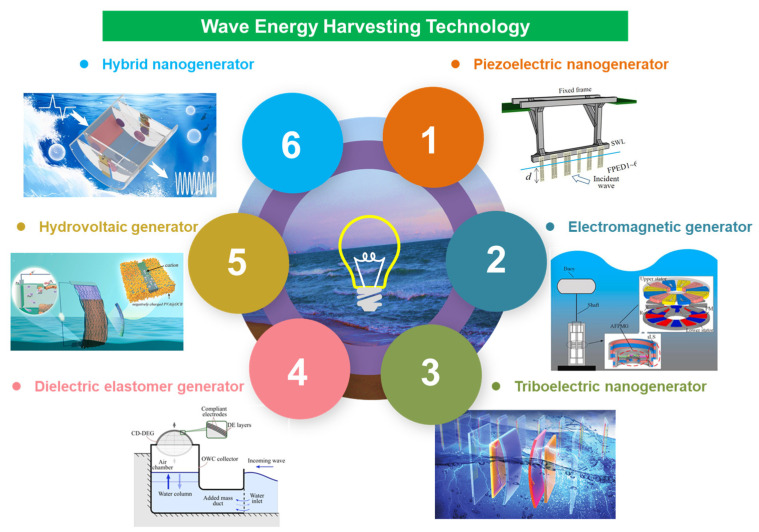
Wave energy harvesting technology based on micro-energy technology. Reprinted with permission from [15,16,17,18,19,20].

This paper provides a comprehensive review of recent advancements in micro-energy-based ocean wave energy harvesting, as depicted in Figure 1. It presents the latest progress in piezoelectric effects [15], electromagnetic effects [16], triboelectric effects [17], dielectric–elastic effects [18], hydrovoltaic effects [19], and hybrid mechanisms [20] for wave energy harvesting while comparing the advantages and disadvantages of each method. Furthermore, it offers insights into the future trajectory of wave energy harvesting technology.

**Piezoelectric nanogenerator (PENG):** Piezoelectric energy harvesting technology utilizes the positive piezoelectric effect of specific materials to efficiently convert mechanical energy into electrical energy. The advantages of piezoelectric nanogenerators lie in their simplistic structure, low thermal output, immunity to electromagnetic interference, ease of processing, and potential for miniaturization and integration. However, these devices exhibit poor low-frequency response characteristics, necessitating larger device sizes for the effective acquisition of low-frequency wave energy.

**Electromagnetic generator (EMG):** Electromagnetic generators are devices that convert mechanical energy into electrical energy based on Faraday’s law of electromagnetic induction. Due to their high energy conversion efficiency, electromagnetic generators are widely employed in large-scale wave energy power stations. However, it is constrained by their bulky size, heavy weight, and susceptibility to electromagnetic interference, thereby limiting the application in small-scale wave energy generation.

**Triboelectric nanogenerator (TENG):** Triboelectric nanogenerators can effectively convert mechanical energy into electrical energy through the principles of triboelectrification and electrostatic induction. As a novel technology for energy harvesting, it offers advantages such as simple structure, compact size, rapid response speed, lightweight design, easy preparation process, wide material selection range, and environmental friendliness. Consequently, it has witnessed rapid development in the field of wave energy harvesting. However, its current limitations lie in a low output power and poor performance reliability.

**Dielectric elastomer generator (DEG):** The dielectric elastomer generator possesses the advantages of high energy density, extensive deformation capacity, simple structure, and low cost. However, it necessitates an external high-voltage bias power supply. To address the issue of relying on an external high-voltage bias power supply, research projects are inclined toward employing self-bias; nevertheless, this necessitates an intricate circuit design. Furthermore, the materials are susceptible to micro-cracks during substantial deformation, thereby impacting its reliability.

**Hydrovoltaic generator:** The hydrovoltaic generator utilizes the direct interaction between nanomaterials and water to generate electricity, offering the advantages of cost-effectiveness, controllable performance, and the absence of additional mechanical energy input. It represents a promising energy conversion device with significant development potential. However, its efficiency is relatively modest while facing strong environmental opposition. Currently, it remains in the laboratory research stage and poses challenges for designing large-scale arrays.

**Hybrid nanogenerator:** The combination of various energy conversion principles and technologies enables composite energy capture devices to effectively enhance the efficiency and scope of energy harvesting from the environment. Although in certain cases this may lead to a reduction in performance indicators for individual technologies, overall, it provides more options for energy capture and a higher utilization efficiency.

## 2. Piezoelectric Nanogenerator

The utilization of piezoelectric nanogenerators represents a prominent approach for the conversion of mechanical energy into electrical energy [21,22,23]. The piezoelectric nanogenerator utilizes the piezoelectric effect to convert mechanical energy into electrical energy [24]. It generates electrical energy through the stress deformation of piezoelectric materials in various manners, exhibiting advantages such as a high power density, a compact size, and a simplistic structure [25]. The piezoelectric wave energy acquisition device can be categorized into two modes: direct coupling and frequency amplification.

### 2.1. Direct-Coupled Type

**Figure 2:**
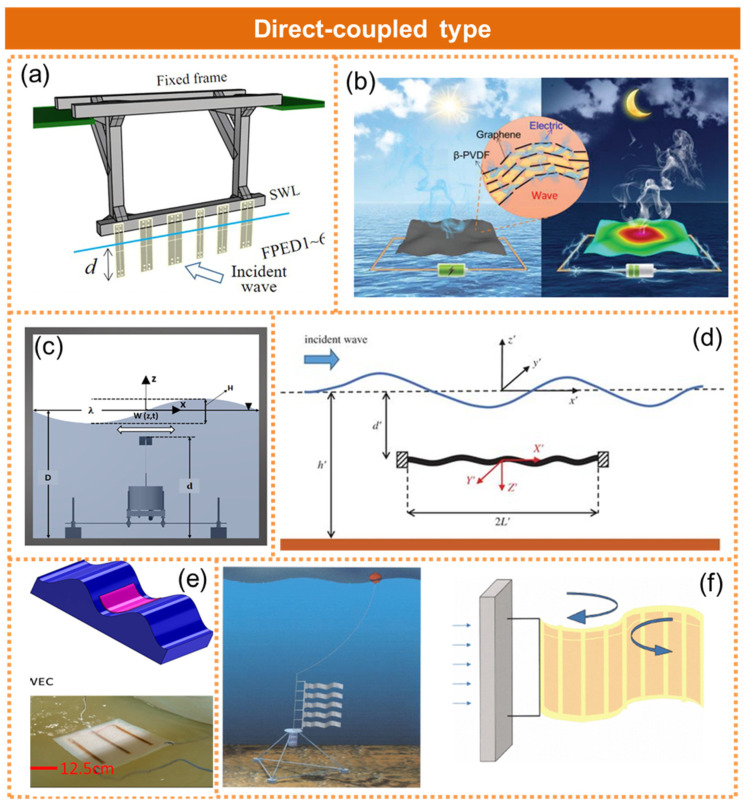
The piezoelectric nanogenerator in the form of direct coupling to ocean waves. (**a**) A flexible device coating with piezoelectric paint for harvesting wave energy. *Ocean Engineering*, reprinted with permission from [15]. (**b**) A multifunctional system capable of both power generation and seawater desalination. Reprinted with permission from [26]. (**c**) Waterproof piezoelectric wave energy harvester. Reprinted with permission from [27]. (**d**) A novel wave energy conversion device consisting of twin piezoelectric wafers that were flexibly clamped at both ends. Reprinted with permission from [28]. (**e**) A buoyant water wave energy converter by integrating a piezoelectric fiber composite. Reprinted with permission from [29]. (**f**) An eel-inspired current energy harvesting device. Reprinted with permission from [30].

The direct contact type involves the direct interaction between the piezoelectric material and the water wave, enabling resonance power generation. This approach primarily utilizes flexible piezoelectric materials that can be directly affixed to the water surface and oscillate in sync with the wave. Mutsuda et al. proposed a flexible piezoelectric device for the efficient conversion of wave energy into electrical energy by utilizing a laminated structure comprising elastic materials and piezoelectric coatings, which can deform under the impact of waves, as illustrated in Figure 2a [15]. Huang et al. utilized polyvinylidene fluoride (PVDF) in conjunction with graphene to develop a multifunctional system capable of both power generation and seawater desalination, as shown in Figure 2b [26]. The incorporation of graphene induced a phase transition in the PVDF crystal, converting it from the α phase to the piezoelectric self-assembled β phase. The resulting film effectively converted wave energy into electricity, exhibiting an output voltage of 2.6 V (±1.3 V) and an energy density of 2.11 Wm^−2^ at an excitation frequency of 1 Hz. Kazemi et al. developed a wave energy collector that utilized waterproof piezoelectricity to harness energy from the longitudinal and lateral motion of ocean waves, as depicted in Figure 2c [27]. The precise waterproof design enabled the complete submergence of the piezoelectric cantilever, thereby enhancing its collection efficiency. Renzi developed a novel wave energy conversion device consisting of twin piezoelectric wafers that were flexibly clamped at both ends and compelled to oscillate in response to incoming sea surface waves, as depicted in Figure 2d [28]. The series-connected piezoelectric ceramic layers effectively transformed the plate’s elastic motion into valuable electricity through the utilization of the piezoelectric effect. Kordmahale et al. developed a buoyant water wave energy converter by integrating a piezoelectric fiber composite (MFC) with an economical elastomer, as shown in Figure 2e [29]. Its performance was evaluated in a water tank, demonstrating an output power of 29.7 µW under low-frequency water waves below 2 Hz, thereby showcasing its remarkable energy conversion efficiency at lower frequencies. Furthermore, it can be conveniently affixed to or submerged in water. Taylor et al. devised an eel-inspired current energy harvesting device, which harnessed eddy currents generated behind a blunt body to induce deformation in piezoelectric elements, thereby facilitating the conversion of mechanical flow energy in oceans and rivers into electricity, as illustrated in Figure 2f [30].

### 2.2. Frequency Amplification

**Figure 3:**
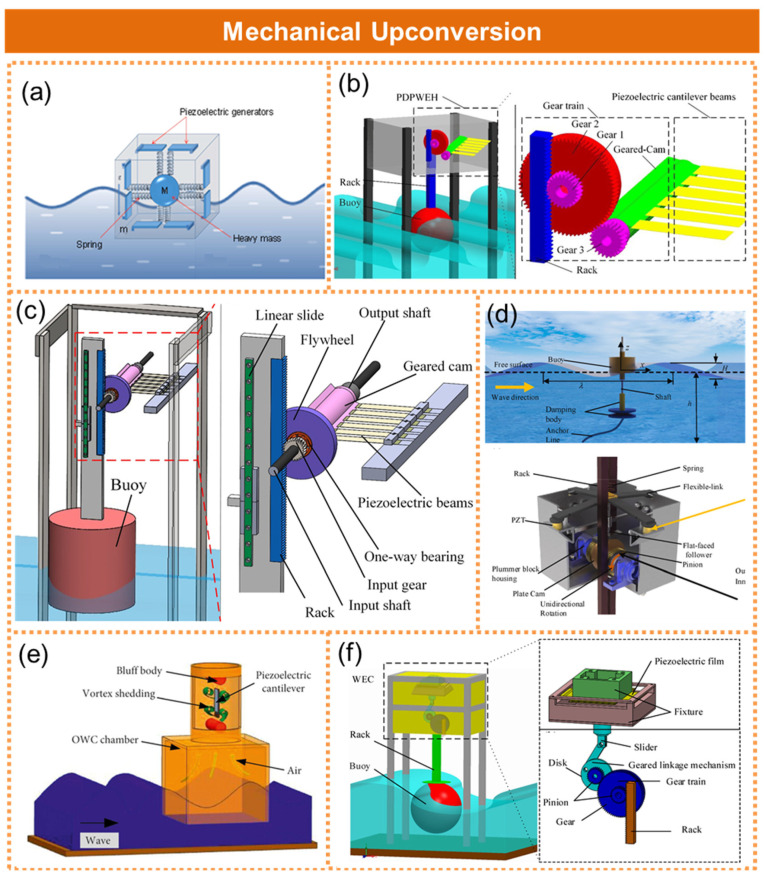
The piezoelectric nanogenerator with mechanical upturn frequency for wave energy acquisition. (**a**) A compact piezoelectric wave energy harvester based on the principle of acceleration-driven mechanism. Reprinted with permission from [31]. (**b**) A piezoelectric wave energy harvester using plucking-driven and frequency up-conversion mechanism energies. Reprinted with permission from [32]. (**c**) A one-way plucking-driven piezoelectric wave energy harvester. Reprinted with permission from [33]. (**d**) A multi-column piezoelectric lamella oscillating floating body wave energy harvesting device. Reprinted with permission from [34]. (**e**) Enhancement of the piezoelectric cantilever beam performance via vortex-induced vibration. Reprinted with permission from [35]. (**f**) A piezoelectric wave-energy converter equipped with a geared-linkage-based frequency up-conversion mechanism. Reprinted with permission from [36].

The utilization of up-frequency technology enables the conversion of low-frequency wave energy into high-frequency vibrations, thereby enhancing the efficiency of the energy conversion. The two commonly employed up-conversion modes are mechanical up-conversion and magnetic drive up-conversion. Cai et al. proposed a compact piezoelectric wave energy harvester based on the principle of an acceleration-driven mechanism. The experimental results demonstrated that the solitary piezo-wave energy collector can achieve an output power of 5 mW, as depicted in Figure 3a [31]. The piezo-wave energy collector designed by Feng et al. incorporated a toggle drive and frequency up-conversion mechanism, as illustrated in Figure 3b [32]. The collector utilized a gear mechanism to amplify the frequency of the water wave oscillation. It possessed a maximum output voltage of 12.4 V, a maximum RMS voltage of 5.01 V, and a maximum average electrical power of 125.5 μW. Chen et al. developed a one-way plucking-driven piezoelectric wave energy harvester, as demonstrated in Figure 3c [33]. The device was comprised of a buoyant cylindrical buoy, an up-conversion mechanism employing a unidirectional bearing, and a piezoelectric element consisting of an array of composite cantilever beams with embedded piezoelectric bodies. The unidirectional bearing converted the cyclic clockwise and counterclockwise rotations of the input shaft into a singular clockwise rotation of the output shaft. Du et al. proposed a multi-column piezoelectric lamella oscillating floating body wave energy harvesting device, which employed a unique rack, pinion, and CAM system to convert the fluctuating motion of the floating body into a unidirectional rotation. The innovative CAM mechanism achieved frequency control and enhanced the overall efficiency of the energy conversion, as depicted in Figure 3d [34]. Du et al. improved the vibration performance and efficiency of piezoelectric energy harvesting by leveraging the eddy current effect in the air passage, as illustrated in Figure 3e [35]. The high-frequency vortex shedding vibration of the piezoelectric cantilever beam was induced by fluid blockage in the outlet channel above the gas chamber, thereby enhancing conversion efficiency. Chen et al. developed a piezoelectric wave energy converter comprising a buoy, a frequency conversion mechanism, and a piezoelectric generator, as depicted in Figure 3f [36]. The variable frequency mechanism consisted of a gear train and a gear-connecting rod mechanism that converted low-frequency fluctuations into high-frequency mechanical movements. In contrast to the fluctuations, the slider exhibited six cycles of displacement and was utilized to excite components for piezoelectric power generation. As a result, the operating frequency of the piezoelectric generating element was six times higher than the frequency of the wave movement.

**Figure 4:**
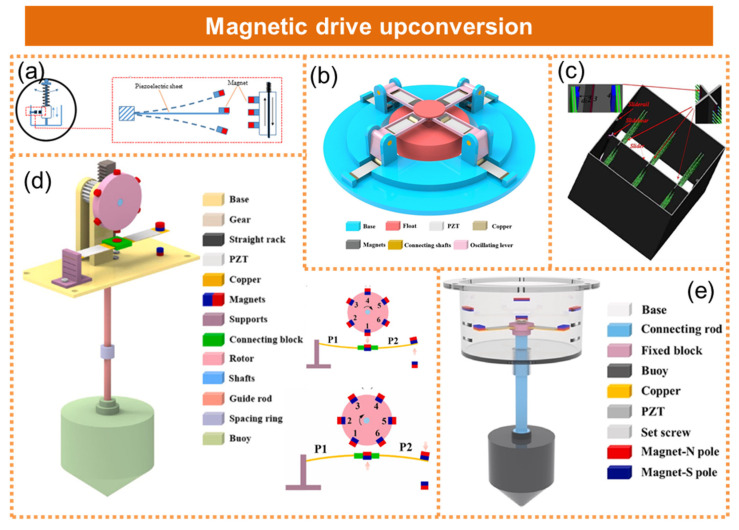
Piezoelectric nanogenerator based on magnetic drive upturn frequency. (**a**) A contactless coupled pendulum and piezoelectric wave energy harvester. Reprinted with permission from [37]. (**b**) A lever-type piezoelectric wave energy harvester based on magnetic coupling and inertial vibration. Reprinted with permission from [38]. (**c**) The magnet increases the vibration frequency of the piezoelectric nanogenerator. Reprinted with permission from [39]. (**d**) A low-frequency piezoelectric wave energy harvester based on segmental beam and double magnetic excitation. Reprinted with permission from [40]. (**e**) A novel piezoelectric wave energy harvester based on cylindrical-conical buoy structure and magnetic coupling. Reprinted with permission from [41].

The resonant frequency of the piezoelectric generator can be altered through the application of magnetic force, thereby demonstrating the technique of magnetic augmentation. Feng et al. utilized a spring pendulum-coupled magnetic drive with up-frequency technology to achieve a 3.8-fold increase in the frequency of the low-frequency wave energy, as shown in Figure 4a [37]. The average output power of each piezoelectric plate was enhanced by 2.4 times, enabling a single piezoelectric plate to generate an average power of 10 milliwatts. The lever-type pressure wave energy collector proposed by Liu et al. was based on magnetic coupling and inertial vibration, which enhanced the output performance through magnetic coupling and the excitation of inertial vibration, as demonstrated in Figure 4b [38]. Under optimal test conditions, the device achieved an impressive output voltage of 36.93 V and an output power of 16.72 mW at a resistance of 6 kΩ. Xie et al. proposed an innovative approach to enhance the efficiency of energy harvesting in piezoelectric wave collectors by incorporating a magnetic block to amplify the oscillator’s speed, as illustrated in Figure 4c [39]. Liu et al. proposed a low-frequency piezo-wave energy collector based on a segmented beam and double magnetic excitation, which employed mechanical and magnetic frequency tuning to enhance output performance in low-frequency wave environments, as shown in Figure 4d [40]. The introduction of a finely tuned magnet enabled the segmented beam to undergo dual magnetic excitation, thereby facilitating deformation. Furthermore, the vibration frequency band was expanded through the excitation of multiple magnets on the rotor. He et al. proposed a novel cylindrical–conical buoy structure-based piezo-wave energy collector with magnetic coupling, where the oscillation of the wave drove the buoy and induced the deformation of the piezoelectric patch under a magnetic force, facilitating efficient energy harvesting from ocean waves. By employing magnetic coupling, low-frequency fluctuations can be converted into high frequency vibrations of the piezoelectric patch, thereby enhancing device performance, as depicted in Figure 4e [41].

## 3. Electromagnetic Generator

The conversion of wave energy into electrical energy in electromagnetic generators is accomplished through the principle of electromagnetic induction, which plays a pivotal role in large-scale marine power stations [42,43,44,45]. However, large-scale power generation equipment encounters the following bottlenecks: insufficient electricity generation from small waves and susceptibility to damage from large waves; exorbitant construction and maintenance costs; challenges in achieving a physical supply of small-scale marine equipment. Consequently, the utilization of micro-energy technology in electromagnetic wave power generation has emerged as a prominent area of research in recent years [46,47,48]. The primary research focus of the electromagnetic wave energy generator lies in enhancing the response characteristics of the device under low-frequency wave energy excitations.

**Figure 5:**
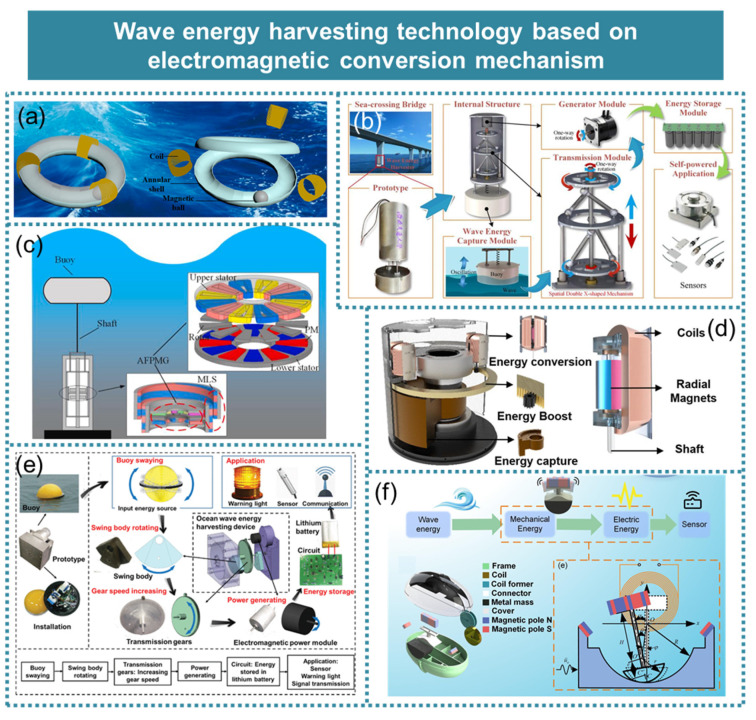
Wave energy harvesting technology utilizing electromagnetic generator. (**a**) A ring electromagnetic generator. Reprinted with permission from [48]. (**b**) A wave energy harvesting system for oscillating buoys based on a spatially double X-shaped mechanism. Reprinted with permission from [49]. (**c**) Maximum power point tracking control based on inertial force for underwater direct-drive wave energy converter. Reprinted with permission from [16]. (**d**) An integrated wave energy harvesting system that exhibited both omnidirectionality and high efficiency. Reprinted with permission from [50]. (**e**) An electromagnetic wave energy harvester based on a highly efficient pendulum mechanism. Reprinted with permission from [47]. (**f**) An electromagnetic generator incorporating a Halbach array. Reprinted with permission from [51].

The ring electromagnetic generator proposed by Wang et al. demonstrated efficient wave energy harvesting capabilities [48], as shown in Figure 5a. When subjected to an external excitation frequency of 1.8 Hz, a single group of coils achieved a maximum open circuit voltage of 7.2 V and a short circuit current of 21.2 mA. Furthermore, when connected to an external load of 150 Ω, the generator exhibited an impressive output power of 80.87 mW. Xie et al. proposed a wave energy harvesting system for oscillating buoys based on a spatially double X-shaped mechanism [49], as demonstrated in Figure 5b. The system comprised four modules: a wave energy capture module, a transmission module, a generator module, and an energy storage module. The transmission module converted the vertical motion of the buoy into the unidirectional rotational motion of the generator input shaft in the generator module to generate electrical energy. When subjected to 1.2 Hz frequency and 15 mm amplitude wave excitation, the system voltage can reach approximately 3 V. The proposed underwater direct drive wave energy converter of Li et al. introduced the integration of an inertial force with the electromagnetic force, facilitating adjustments to the magnetic pole position as well as enhancing dynamic response performance [16], as illustrated in Figure 5c. The findings illustrated that utilizing maximum power tracking control based on inertial force enabled the optimal utilization of wave energy and expanded the scope of power capture. Li et al. proposed an integrated wave energy harvesting system that exhibited both omnidirectionality and high efficiency [50], as shown in Figure 5d. By synergistically combining the design of a chaotic pendulum with an electromagnetic power generation mechanism, they successfully achieved an exceptional output power of 520 mW under ultra-low-frequency wave excitation conditions characterized by a wave height of 20 cm and a period of 1 s. Li et al. proposed an electromagnetic wave energy harvester based on a highly efficient pendulum mechanism [47], as depicted in Figure 5e. The pendulum effectively detected ultra-low-frequency fluctuations and drove the rotor of the electromagnetic power module to rotate at a high speed through the transmission gear system. This device was deployed within an ocean buoy and underwent testing in the Yellow Sea. Under peak wave conditions exceeding 0.6 m, it achieved a maximum peak-to-peak output voltage of 15.9 V. Pan et al. developed an electromagnetic generator incorporating a Halbach array, inspired by a roller design, to enhance the performance of low-frequency wave energy harvesting [51], as illustrated in Figure 5f. Sea trials were conducted in the East China Sea, demonstrating the effective operation of the device in real ultra-low-frequency (<1 Hz) wave conditions with a maximum peak power output of 120 mW.

**Figure 6:**
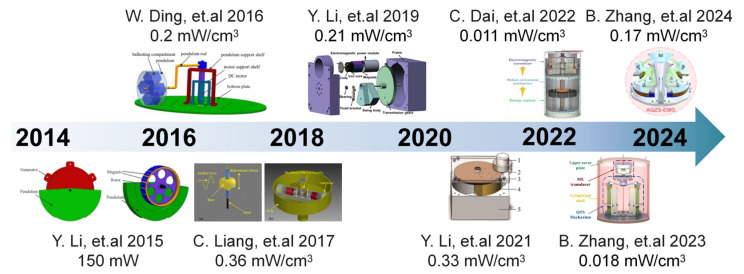
The development of EMG-based wave energy harvesting devices. Reprinted with permission from [46,47,52,53,54,55,56,57].

The design and manufacturing technology of electromagnetic generators has been continuously improved with the advancement of science and technology, enabling them to achieve miniaturization while maintaining high efficiency [46,47,52,53,54,55,56,57]. Figure 6 presents an overview of the advancements made in wave energy harvesting pertaining to miniaturized electromagnetic generators. The researchers Ding et al. developed a low-frequency horizontal pendulum electromagnetic marine kinetic energy collector specifically designed for underwater mooring platforms [52]. This energy harvester allowed for the precise adjustment of the natural frequency by altering the pitch angle of the pendulum to match the dominant excitation frequency. The experimental results demonstrated that the maximum average output power can reach 0.3 W, while achieving a normalized power density of 3453.8 kg/m^3^. Liang et al. proposed a power output system based on a mechanical motion rectifier (MMR) and designed a prototype for a wave energy converter [53]. The power output system utilized the integration of two one-way bearings into the rack and pinion system to convert bi-directional wave motion into the unidirectional rotation of the generator. Mechanical energy was then efficiently converted into electrical energy by an electromagnetic generator with an impressive power density of up to 0.36 mW/cm^3^. The wave energy harvesting system proposed by Dai et al. for an unmanned craft was based on bi-wing flywheels [55]. A screw nut mechanism and a double-wing flywheel mechanism were employed to convert the oscillating vibration of the mass block into the relative unidirectional rotation of the magnet flywheel and coil flywheel. The electromagnetic energy exchange module converted the mechanical energy of the flywheel into electrical energy, which was then transmitted to the circuit module. Compared to energy harvesting equipment without a mechanical motion rectifier, this system achieved a 51.64% increase in energy harvesting power. Zhang et al. introduced a tunable zero-stiffness electromagnetic generator (EMG) designed for the efficient extraction of ultra-low-frequency vibration energy from ocean waves [57]. Through the implementation of a tilting pendulum design, the natural frequency of the EMG can be effectively reduced and finely adjusted to meet both ultra-low-frequency and broadband requirements. At an excitation frequency ranging from 0.5 to 1.1 Hz, the EMG demonstrated its potential by achieving a maximum output power of 32.01 mW, highlighting its capability in harnessing clean blue energy.

## 4. Triboelectric Nanogenerator

Triboelectric nanogenerators (TENGs) utilize the principles of triboelectrification and electrostatic induction to efficiently convert mechanical energy into electrical energy [58,59,60,61,62]. Under low-frequency operating conditions, the triboelectric nanogenerator (TENG) exhibits notable advantages in terms of high energy conversion efficiency, elevated power density, lightweight design, and cost-effectiveness [63,64,65,66,67]. With continuous research advancements, the TENG has gained extensive utilization in the realm of blue energy and has achieved remarkable progress [68,69,70,71,72]. The wave energy harvesting methods of the TENG can be categorized into two primary forms: solid–solid friction and solid–liquid friction.

### 4.1. Solid–Solid Friction

**Figure 7:**
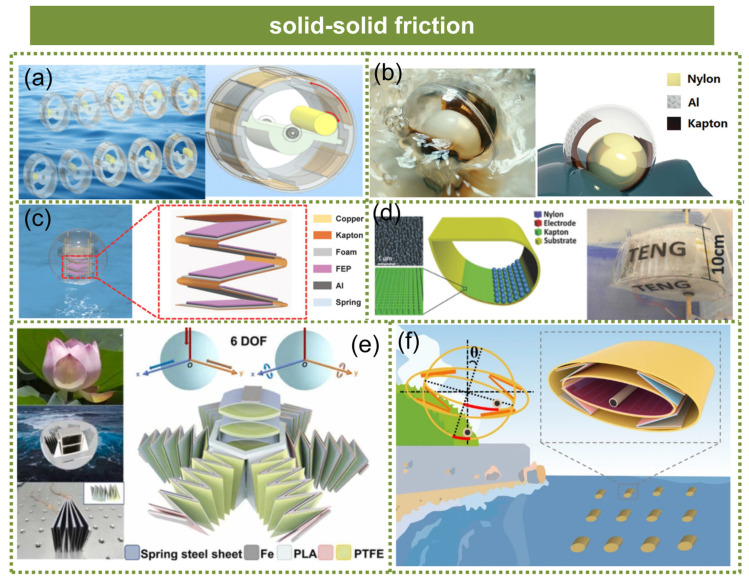
The technology for harvesting wave energy through solid–solid friction. (**a**) A high-performance self-powered oil spill positioner utilizing a soft contact triboelectric nanogenerator. Reprinted with permission from [73]. (**b**) A triboelectric nanogenerator based on fully enclosed rolling spherical structure. Reprinted with permission from [74]. (**c**) Spherical triboelectric nanogenerators based on spring-assisted multilayered structure. Reprinted with permission from [75]. (**d**) A duck-shaped triboelectric nanogenerator. Reprinted with permission from [78]. (**e**) Flower-like triboelectric nanogenerator. Reprinted with permission from [77]. (**f**) A fully symmetric TENG with an elliptic cylinder structure. Reprinted with permission from [78].

The majority of solid–solid TENGs are predicated on enclosed structures. Li et al. developed a high-performance self-powered oil spill positioner utilizing a soft contact triboelectric nanogenerator (SC-TENG) [73], as shown in Figure 7a. To achieve soft contact, rabbit hair was attached to the rotor, effectively reducing friction resistance, facilitating a rapid charge transfer to the electrode, and enhancing component durability. Wang et al. proposed a self-contained and fully enclosed TENG that incorporated a rolling ball within a swinging spherical housing [74], as demonstrated in Figure 7b. When subjected to the excitation of water waves, this spherical TENG with a diameter of 6 cm can generate an instantaneous output power of up to 10 mW. The TENG array designed by Tian et al. comprised spherical TENG units based on a spring-assisted multilayer structure for the efficient capture of water wave energy [75], as illustrated in Figure 7c. The incorporation of a spring structure enhanced the output performance of the spherical TENG by converting low-frequency water wave motion into high-frequency vibration, while the utilization of a multi-layer structure maximized the space efficiency, resulting in increased output power from each spherical unit. Ahmed et al. proposed a fully enclosed duck-shaped triboelectric nanogenerator (TENG) that demonstrated efficient energy harvesting from random and low-frequency water waves [76], as shown in Figure 7d. By conducting a fluid–structure coupling analysis, the device’s exceptional dynamic performance, mechanical efficiency, and stability under water wave conditions were substantiated. Inspired by biological structures, various devices incorporating biomimetic designs have been developed to enhance the efficiency of energy capture. Wen et al. devised and fabricated a flower-like triboelectric nanogenerator (TENG) capable of harvesting kinetic energy from water waves with six degrees of freedom [77], as depicted in Figure 7e. By leveraging the “flowering” and “folding” motions, this device effectively converted the kinetic energy of water waves into electrical energy. Tan et al. developed a fully symmetric TENG with an elliptic cylinder structure, specifically designed for all-weather blue energy harvesting to prevent overturning [78], as illustrated in Figure 7f. The novel elliptic cylindrical structure exhibited exceptional inherent stability, heightened sensitivity, and notably distinctive resistance against overturning for the TENG.

### 4.2. Solid–Liquid Friction

Solid–liquid TENGs (SL-TENGs) represent a novel technology that integrates contact electrification (CE) and electrostatic induction to harness renewable energy stored in natural water sources [79,80,81]. Owing to their distinctive advantages, including high energy density, versatile material options, and the potential for large-scale deployment, SL-TENGs have garnered increasing attention in recent years [82,83,84]. Numerous studies have demonstrated their significant potential in the field of wave energy harvesting.

**Figure 8:**
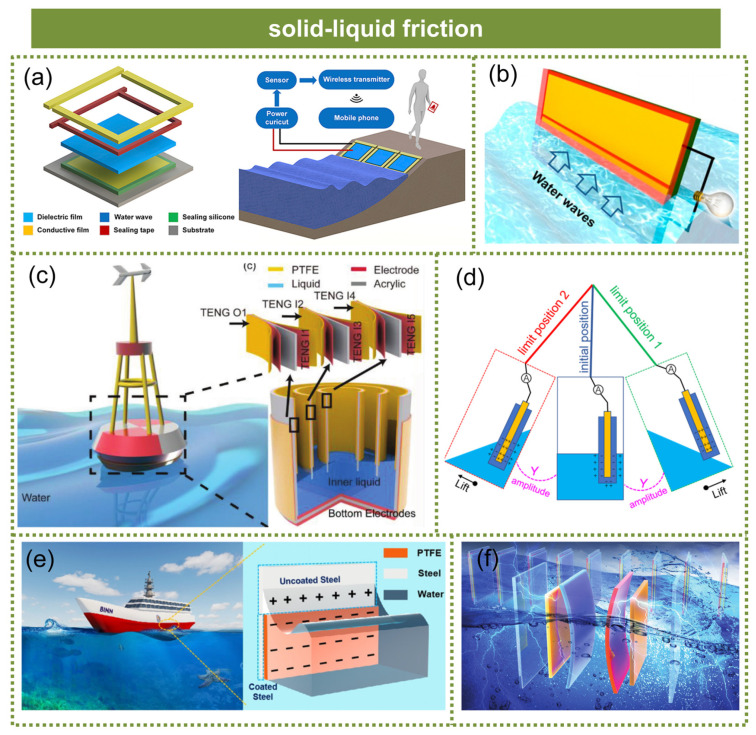
The technology for harvesting wave energy through solid–liquid friction. (**a**) A liquid–solid contact triboelectric mechanism-based thin film. Reprinted with permission from [85]. (**b**) A liquid–solid band tribogenerator based on fluorinated ethylene propylene film. Reprinted with permission from [86]. (**c**) A buoy-like solid–liquid TENG with a lifebuoy structure. Reprinted with permission from [87]. (**d**) A novel solid–liquid TENG based on vortex-excited resonance. Reprinted with permission from [88]. (**e**) A seawater-based TENG for marine anticorrosion. Reprinted with permission from [89]. (**f**) A liquid–solid TENG arrays based on dynamic electric-double-layer. Reprinted with permission from [17].

Liu et al. proposed a solid–liquid contact triboelectric mechanism-based thin film blue energy collector, which incorporated a novel external cylindrical electrode comprising a strip electrode (B electrode) and a U-shaped electrode (U electrode). This design effectively mitigated the shielding effect against water, thereby significantly enhancing the output performance [85], as shown in Figure 8a. Zhu et al. proposed a solid–liquid band TENG based on a fluorinated ethylene propylene film [86], as demonstrated in Figure 8b. The utilization of the triboelectric effect at the solid–liquid interface, devoid of any mechanical components, successfully demonstrated a practical and technologically advanced approach for harnessing wave energy. Li et al. proposed a buoy-like solid–liquid TENG with a lifebuoy structure, which effectively harnessed energy from diverse wave vibrations and exhibited an output 48.7 times higher than that of a solid–solid TENG with an equivalent surface area [87], as illustrated in Figure 8c. Li et al. proposed a novel solid–liquid TENG based on vortex-excited resonance [88], as shown in Figure 8d. The experimental results demonstrated the successful conversion of vortex energy into electrical energy by the device, achieving maximum output voltage in a resonant state. These findings highlighted its significant potential for “blue energy” harvesting. Zhang et al. proposed a seawater-based TENG for marine anticorrosion, which demonstrated exceptional performance through the judicious selection of dielectric film materials [89], as depicted in Figure 8e. Moreover, when compared to commercial anticorrosion coatings, the device exhibited a 43.8% reduction in friction coefficient with seawater, thereby facilitating decreased navigation resistance for ships. Liang et al. proposed solid–liquid TENG arrays based on a dynamic electric-double-layer [17], as illustrated in Figure 8f. The TENG array demonstrated the desired output performance under irregular water waves, yielding a stable output current of 60.0 µA, an output voltage of 60.0 V, and an average power density of 5.38 W m^−3^.

**Figure 9:**
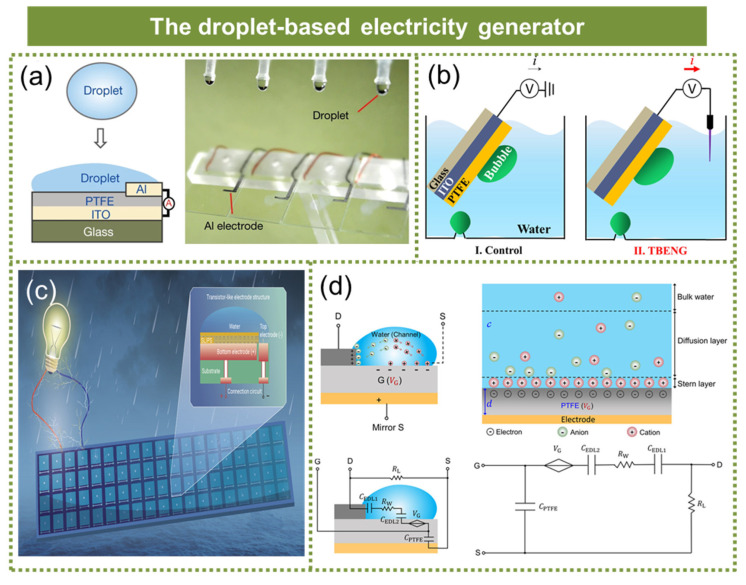
The droplet-based electricity generator. (**a**) A droplet generator resembling the structure of a field-effect transistor. Reprinted with permission from [90]. (**b**) A transistor-inspired bubble energy generator. Reprinted with permission from [91]. (**c**) A lubricant-protected transistor-like generator. Reprinted with permission from [92]. (**d**) A systematic design strategy for a volume-effect droplet generator. Reprinted with permission from [93].

The droplet-based electricity generator is a prototypical solid–liquid TENG. However, the exploitation of water energy stored in raindrops poses challenges for traditional hydroelectric power generation technology due to its low frequency, sparse distribution, and varying sizes. In 2020, Xu et al. devised a droplet generator resembling the structure of a field-effect transistor, which successfully overcame the historical research bottleneck associated with a low power density in droplet generators, as shown in Figure 9a [90]. The maximum energy generated per square meter can reach 50.1 W. In 2022, Yan et al. developed a transistor-inspired bubble energy generator for the direct and efficient harvesting of energy from small bubbles, as illustrated in Figure 9b [91]. By synergistically regulating the surface wetness and transistor electrode, they effectively promoted the rapid diffusion and departure of bubbles, transforming the initial liquid–solid interface into a gas–solid interface under bubble gating. As a result, the output performance was improved by at least one order of magnitude. In 2022, Song et al. proposed a design for a lubricant-protected transistor-like generator (LA-TEG), as illustrated in Figure 9c [92]. This innovative design demonstrated excellent and stable power generation performance across various challenging environments, including low temperatures, high humidity levels, and elevated salinity conditions. Leveraging advanced printed circuit board technology, the design significantly reduced the number of circuit nodes and connections while offering exceptional scalability for large-scale array integration. Zhang et al. proposed a systematic design strategy for a volume-effect droplet generator in order to achieve an ultra-high instantaneous output and extremely short charging time [93]. This is demonstrated in Figure 9d. By optimizing the dielectric layer thickness, droplet ion concentration, and external load, the bulk effect droplet generator can maintain stable output at 80% of its optimal performance after approximately 30 droplets impacted within a span of 10 s. The generator exhibited an instantaneous power density of 2.03 kW/m^2^ and an average power density per pulse of 357 W/m^2^.

**Figure 10:**
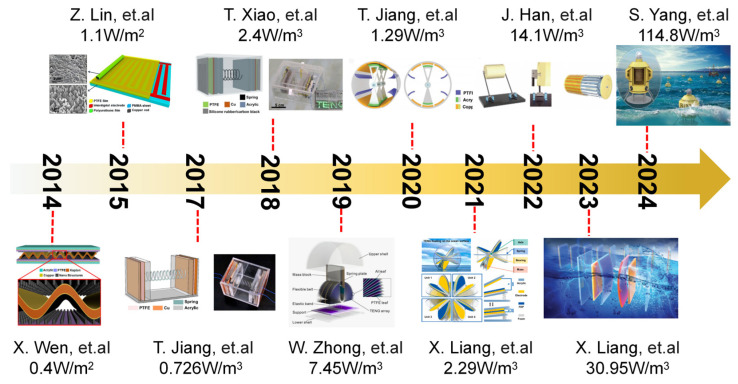
The development of TENG-based wave energy harvesting devices. Reprinted with permission from [7,17,94,95,96,97,98,99,100,101].

After extensive research and exploration conducted by researchers, there has been a significant advancement in the development of wave energy harvesting devices based on TENGs over the past decade [7,17,94,95,96,97,98,99,100,101]. Consequently, we have compiled a comprehensive overview of this progress, as depicted in Figure 10.

The TENG designed by Wen et al. in 2014, based on a wave structure, enabled the acquisition of wave energy through device packaging [7]. Despite an output power of only a few tenths of a watt per cubic meter, this research paved the way for utilizing nanomaterials to harness renewable energy from the ocean. In 2018, Tian et al. developed a TENG featuring a silicone rubber/carbon black composite electrode to efficiently convert water wave energy into electricity [96]. The utilization of silicon-based electrodes with a soft texture enabled enhanced contact with the dielectric film, while the incorporation of a spring structure facilitated the conversion of low-frequency water wave motion into high-frequency vibrations. Through the meticulous optimization of the triboelectric material pair and friction surface area, the device achieved an output power exceeding watts per cubic meter. In 2020, Jiang et al. proposed an oscillating structure triboelectric nanogenerator with high energy conversion efficiency for ultra-low-frequency water wave energy harvesting [98]. The device’s ruggedness and durability were enhanced through the design of the air gap and flexible brush, resulting in minimal friction resistance and sustainable triboelectric charge generation. This research offered a promising solution for large-scale blue energy harvesting. In 2024, Yang et al. successfully integrated the oscillating water column (OWC) wave energy conversion mechanism with triboelectric nanogenerator (TENG) technology for the first time, resulting in a non-contact turntable structure specifically designed for OWC-TENG systems [101]. The device achieved an impressive output power density exceeding 100 W per cubic meter, thereby providing a reliable and stable power supply for small sensors deployed at sea. This groundbreaking work offered a novel solution for efficiently harnessing low-frequency micro-amplitude wave energy and supplying power to various offshore equipment.

## 5. Dielectric Elastomer Generator

The dielectric elastomer generator (DEG) is a capacitive solid-state device that utilizes highly stretchable dielectrics and conductors to efficiently convert mechanical energy into a high-voltage direct current [102,103,104]. Its exceptional performance in terms of energy conversion and power density has been extensively demonstrated through quasi-static experimental tests with specified deformations.

**Figure 11:**
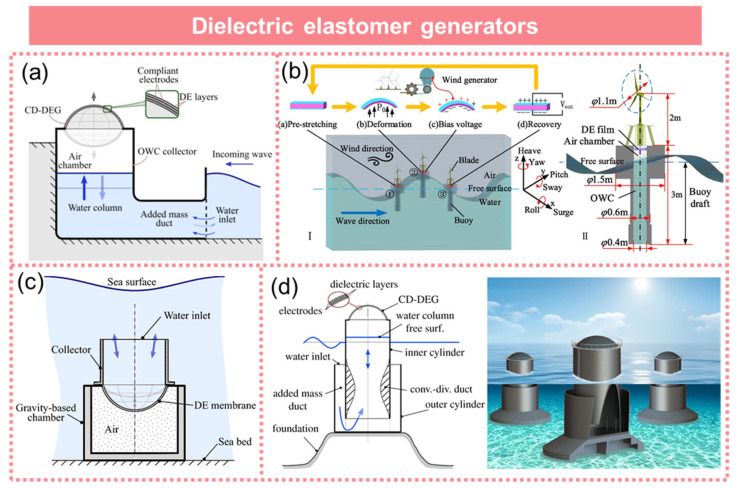
Dielectric elastomer generator. (**a**) Resonant wave energy harvester based on dielectric elastomer generator. Reprinted with permission from [19]. (**b**) A dielectric elastomer wave energy collector integrated with an auxiliary wind turbine. Reprinted with permission from [105]. (**c**) A broad-banded pressure differential wave energy converter based on dielectric elastomer generators. Reprinted with permission from [106]. (**d**) The DEG based on the integration of nonlinear potential fluid dynamics and electrohyperelastic theory. Reprinted with permission from [107].

Giacomo Moretti et al. proposed a DEG-based wave energy harvesting structure for the conversion of wave energy into electrical power [19], as shown in Figure 11a. Under resonant conditions, the device exhibited a peak power output of 0.87 W per cycle, while achieving an impressive wave energy conversion efficiency of 18%. Du et al. proposed a dielectric elastomer wave energy collector integrated with an auxiliary wind turbine for self-bias voltage generation, resulting in significantly enhanced electromechanical conversion efficiency [105], as demonstrated in Figure 11b. Righi et al. proposed a novel differential pressure wave energy converter featuring a direct contact between a DEG and seawater, thereby introducing an innovative concept to harness wave energy [106], as illustrated in Figure 11c. The experimental findings demonstrated the exceptional output performance of this device across diverse sea conditions. The DEG was designed by Moretti et al. through the integration of nonlinear potential fluid dynamics and electrohyperelastic theory [107], as depicted in Figure 11d. The experimental results demonstrated a peak power output of up to 3.8 W for this device.

The Stanford Research Institute (SRI) first proposed dielectric elastomer materials in 1991, and after over three decades of development, it has been demonstrated that these materials can serve not only as actuation components but also as efficient power generation materials. Table 1 provides a summary of some representative cases showcasing the utilization of dielectric elastomer generators for wave energy harvesting. The dielectric elastomer generator developed by S. Chiba et al. demonstrated the capability to generate 42 mJ of energy per wave cycle with a wave height of 6 cm, as evidenced by experimental results [108]. The self-powered dielectric elastomer nanogenerator, developed by Xu et al., efficiently converted tensile mechanical energy into electrical energy under biaxial tension, eliminating the need for any external power supply [109]. By utilizing the alternating current (AC) method, the device achieved a charge density of 26 mC m^−2^ per mechanical cycle and an impressive energy density of up to 140 mJ g^−1^. Lv et al. conducted an analysis of the constitutive relationships under various hyperelastic models, based on both theoretical and experimental studies [110]. Subsequently, a dielectric elastomer wave energy generator was designed and fabricated through theoretical optimization. The findings demonstrated that the device exhibited a power density of 7.322 J/g. The U-shaped oscillating water column model proposed by Moretti et al. incorporated a dielectric elastomer generator, demonstrating excellent agreement between the model predictions and experimental data [111]. This model was capable of accurately predicting the output characteristics of full-scale U-shaped oscillating water columns with a DEG.

In conclusion, despite the significant advantages of dielectric elastomer generators in energy collection and conversion, they still encounter certain challenges in practical applications, such as enhancing energy collection efficiency, prolonging service life, and simplifying power requirements.

## 6. Hydrovoltaic Generator

The hydrovoltaic generator employs evaporation as a driving force to propel water through functional nanochannels, thereby converting ambient heat energy into electricity [112,113,114,115,116,117]. This novel environmentally friendly technology holds immense potential for widespread application in marine energy harvesting.

**Figure 12:**
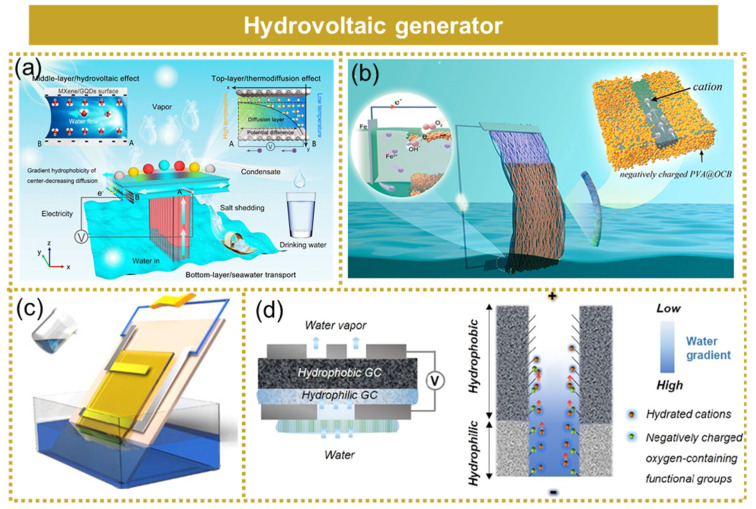
Hydrovoltaic generator. (**a**) A biomimetic interfacial evaporation-driven hydrovoltaic generator. Reprinted with permission from [118]. (**b**) A novel, flexible dual-mode power generator. Reprinted with permission from [18]. (**c**) A straightforward and efficient ion-selective hydrogel film. Reprinted with permission from [119]. (**d**) A novel hydrovoltaic generator utilizing a Janus double-layer membrane with asymmetric wettability. Reprinted with permission from [120].

Chen et al. developed a biomimetic interfacial evaporation-driven hydrovoltaic generator inspired by the lotus plant, which efficiently generated both water vapor and electricity from seawater instead of freshwater [118], as shown in Figure 12a. By emulating the transpiration process of the lotus stem and leaf, photothermal evaporation and power generation were significantly enhanced. The combined effect of thermal diffusion led to an impressive output power density of up to 45.6 µW cm^−2^. The design of a flexible two-mode electric nanogenerator capable of harvesting energy from a dynamically changing aqueous solution was reported by Li et al. In contrast to previous studies, the device was constructed on a highly porous carbon black/PVA film with negatively charged groups, featuring a hydrophobic top and a hydrophilic bottom modification [18], as demonstrated in Figure 12b. The experimental findings demonstrated that the device exhibited sustained performance across more than 10 orders of magnitude in ion concentrations. Pei et al. successfully developed a straightforward and efficient ion-selective hydrogel film capable of harnessing energy from both seawater and salt solutions at an ambient temperature [119], as illustrated in Figure 12c. Remarkably, a single device exhibited an open circuit voltage of 0.16 V and generated a power output of 0.048 μW when tested in authentic seawater. Eun et al. successfully developed a novel hydrovoltaic generator utilizing a Janus double-layer membrane with asymmetric wettability [120], as depicted in Figure 12d. By substituting deionized water with a 0.6 mL NaCl solution (equivalent to seawater concentration), the generated output voltage and current were measured as 0.55 V and 60 μA, respectively. Remarkably, throughout the entire experimental period of 7 days, the asymmetrical wettability of the membrane remained stable, thereby enabling uninterrupted power generation.

The conversion mechanism of a hydrovoltaic generator can be categorized into the flow potential, ion volt effect, upstream proton diffusion, pseudo-flow mechanism, and other related factors.

**Figure 13:**
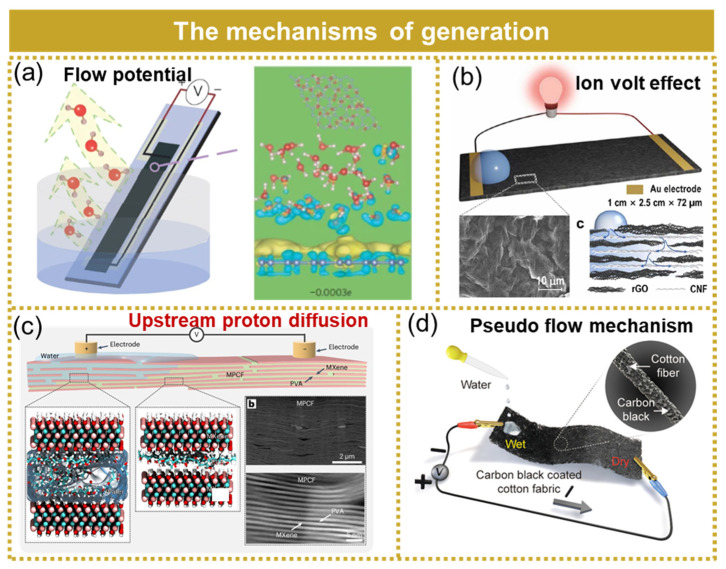
The mechanisms of hydrovoltaic generator. (**a**) Flow potential. Reprinted with permission from [121]. (**b**) Ion volt effect. Reprinted with permission from [122]. (**c**) Upstream proton diffusion. Reprinted with permission from [123]. (**d**) Pseudo-flow mechanism. Reprinted with permission from [124].

**Flow potential:** The voltage generated within the fluid due to the movement of electrolytes through a narrow channel caused by a pressure gradient is referred to as the flow potential. The flow potential, being the first water volt effect studied, efficiently converts differences in hydrostatic pressure into electricity. Xue et al. investigated the current generation mechanism resulting from the evaporation of mesoporous carbon black water using the theory of flow potential, as depicted in Figure 13a [121]. The interstitial space within the mesoporous carbon was considered as the conduit for the water flow, while the aqueous solution was regarded as exhibiting a directional flow during evaporation. Due to the significantly smaller gap spacing compared to the Debye length of the water–solid interface, it can be simplified that water flowed through numerous negatively charged surface micro-channels, leading to flow potential generation and continuous water flow driven by evaporation within these channels, ultimately ensuring uninterrupted electrical energy output.

**Ion volt effect:** The adsorption of ions on the surface of the functional material leads to a modification in carrier concentration, ultimately resulting in the generation of an ion volt effect, which manifests as a potential difference between wet and dry regions. The hydrovoltaic generator proposed by Yu et al. was based on the ion volt effect for energy harvesting in water, as depicted in Figure 13b [122]. This generator consisted of a composite material comprising reduced graphene oxide and cellulose nanofibers. Through optimization, the individual device achieved an output voltage of 0.72 V and a current of 1.2 µA.

**Upstream proton diffusion:** The upstream proton diffusion mechanism of the hydrovoltaic generator is the device that harnesses the power generation mechanism resulting from the counter-directional movement of protons against the flow of water. Xia et al. proposed a hydrovoltaic generator based on the upstream proton diffusion mechanism for harvesting energy from water, as illustrated in Figure 13c [123]. They demonstrated that electricity can also be generated by the movement of protons against the direction of the water flow, known as upstream proton diffusion, within the two-dimensional nanochannel of the MXene/PVA film. When optimized, this device was capable of generating a voltage exceeding 400 mV for over 330 min.

**Pseudo-flow mechanism:** Yun et al. presented a hydrovoltaic generator employing a pseudo-flow mechanism for harvesting water flow energy, as depicted in Figure 13d [124]. The accumulation of protons resulting from the formation of a double-layer at the carbon black/water interface induced a potential difference between the dry and wet sides. The conductive carbon black coating facilitated current conduction through a pseudo-current mechanism. The optimized individual device achieved a peak output voltage of 0.53 V, a maximum output current of 3.91 μA, and an utmost energy density of 1.14 mWh/cm^3^.

## 7. Hybrid Nanogenerator

In early research, the majority of energy harvesting methods employed a single energy conversion mechanism, resulting in low output power and limited energy conversion efficiency within a restricted frequency band range, thus presenting certain limitations. To further enhance the efficiency of energy collection, numerous scholars have started to adopt an approach that combines multiple energy conversion mechanisms [125,126,127,128,129,130,131,132], thereby fully leveraging the complementary advantages offered by these mechanisms to effectively improve both the energy collection efficiency and output performance.

**Figure 14:**
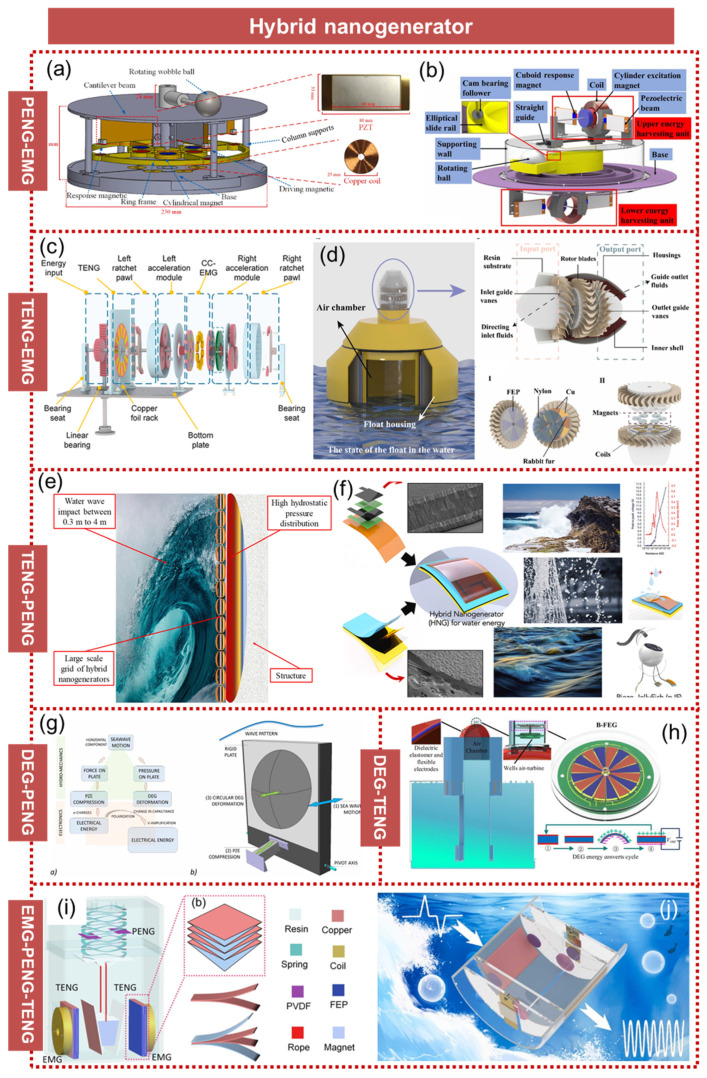
Hybrid nanogenerator. (**a**) A piezo-electromagnetic hybrid wave energy collector driven by a rotating pendulum ball. Reprinted with permission from [133]. (**b**) A mixed-wave vibration energy collector featuring an electromagnetic two-speed mechanism and piezoelectric upturn. Reprinted with permission from [134]. (**c**) A triboelectric–electromagnetic composite-based wave energy harvesting system that is self-powered and self-induced. Reprinted with permission from [135]. (**d**) Hybridized triboelectric–electromagnetic nanogenerator. Reprinted with permission from [136]. (**e**) Synergistic use of triboelectric and piezoelectric phenomena in specific materials. Reprinted with permission from [137]. (**f**) Multifunctional sub-100 µm thickness flexible piezo/triboelectric hybrid water energy harvester. Reprinted with permission from [138]. (**g**) Hybridizing piezoelectric and dielectric elastomer generators. Reprinted with permission from [139]. (**h**) A dielectric elastomer and electret hybrid ocean wave power generator. Reprinted with permission from [140]. (**i**) A broadband hybrid blue energy nanogenerator. Reprinted with permission from [141]. (**j**) Frequency modulated hybrid nanogenerator. Reprinted with permission from [20].

Shi et al. proposed a floating piezo-electromagnetic hybrid wave energy collector driven by a rotating pendulum ball, which converted wave energy into electricity using electromagnetic generators (EMGs) and piezoelectric nanogenerators [133], as shown in Figure 14a. The device employed the frequency up-conversion mechanism to enhance energy conversion efficiency by converting low-frequency vibration energy into high-frequency vibrations. The experimental results demonstrated that the maximum power generated by the proposed hybrid energy collector can reach 21.95 mW at a wave frequency of 1.4 Hz. Jia et al. proposed a mixed-wave vibration energy collector featuring an electromagnetic two-speed mechanism and piezoelectric upturn driven by a rotating ball, aiming to enhance the output voltage through an increased flux change rate in electromagnetic generators (EMGs) and broaden the operating frequency band via bistable motion facilitated by four coupled piezoelectric cantilevers in the piezoelectric nanogenerator [134], as demonstrated in Figure 14b. Zhou et al. proposed a self-powered and self-sensing wave energy harvesting system for smart oceans and cross-sea bridges. The system achieved wave energy acquisition through a coaxial reverse rotating electromagnetic generator. At an excitation of 0.7 Hz and 25 mm, the average power generation of the electromagnetic module can reach 316 mW [135], as illustrated in Figure 14c. The hydrological information of the waves was identified by the triboelectric module, with an amplitude recognition accuracy of 98.17% and a frequency recognition accuracy of 99.33%. Zhang et al. proposed a triboelectric–electromagnetic hybrid nanogenerator (TEH-NG) specifically designed for self-powered ocean buoys [136], as shown in Figure 14d. The buoy incorporated a customized wave energy converter, which efficiently converted wave energy into turbine-mechanical energy by utilizing the pressure difference generated from relative motion. Moreover, the TEH-NG effectively converted this turbine-mechanical energy into electricity. The TENG demonstrated an average power output of 3.40 mW, while the EMG exhibited an average power output of 0.04 W. Jurado et al. successfully harnessed wave energy by synergistically exploiting the triboelectric and piezoelectric phenomena exhibited in specific materials, resulting in a remarkable enhancement of 2.24 and 3.21 times compared to the individual performance of a single triboelectric or piezoelectric nanogenerator [137], as depicted in Figure 14e. Mariello et al. developed a highly adaptable and versatile device for harnessing energy from ocean waves [138], as illustrated in Figure 14f. Through the innovative integration of biocompatible piezoelectric ceramic films with flexible polymer materials, they successfully created a composite device with a width of less than 100 μm. When subjected to an excitation force of 5 N at a frequency of 5 Hz, the power density of the piezoelectric nanogenerator (PENG) reached approximately 6.5 mW m^−2^; meanwhile, the triboelectric nanogenerator (TENG) achieved an impressive power density of 65 mW m^−2^. Boccalero et al. proposed a novel approach for wave energy harvesting by employing piezoelectric elements (PZEs) and dielectric elastomer generators (DEGs) [139], as demonstrated in Figure 14g. The horizontal pressure gradient and water velocity beneath the wave were utilized to compress the PZE and expanded a flexible variable capacitor during each half-wave period, constituting the DEG. The charge generated by the PZE was employed to polarize the DEG, thereby effectively doubling the input energy. Du et al. proposed an innovative oscillating water column energy collector based on dielectric elastomers, coupled with a TENG that provided a bias voltage to the DEG [140], as illustrated in Figure 14h. The novel concept was to utilize the pressure and airflow generated by the motion of the oscillating water column for expanding the DE film, thereby enabling the continuous and synchronous rotation of the rotating generator during each wave cycle. Gao et al. devised an elastic pendulum compound wave energy harvesting device, wherein the design of the elastic pendulum effectively decoupled the collection of horizontal and vertical kinetic energies, thereby enhancing system efficiency [141], as depicted in Figure 14i. Furthermore, by integrating electromagnetic, triboelectric, and piezoelectric conversion mechanisms, the wave energy acquisition capability of the system was enhanced while optimizing the space utilization rate and overall power density. Tian et al. developed a triboelectric–electromagnetic–piezoelectric hybrid energy harvester (TEP-HEH) for low-frequency wave energy harvesting, which incorporated a cantilever beam structure to convert the vibration frequency from 0.2 Hz (analog wave frequency) to 7.2 Hz, significantly enhancing the performance of both the electromagnetic generator (EMG) and piezoelectric nanogenerator (PENG) modules [20], as shown in Figure 14j. By expanding the frequency bandwidth of wave energy collection, this device achieved a power density of up to 5.73 W m^−3^.

The advancements of hybrid nanogenerators in recent years are comprehensively summarized in the Table 2 [142,143,144,145,146,147,148,149,150]. The piezoelectric electromagnetic hybrid energy collector (PEHEH) was proposed by He et al. in 2024 for the purpose of capturing low-frequency wave motion and monitoring the wave environment through self-sensing capabilities [142]. The piezoelectric unit served as the power supply component, while the electromagnetic unit functioned as the self-sensing element, enabling the real-time measurement of the wave amplitude in practical wave environments. The obtained results validated that this hybrid generator possessed both self-supplying and self-sensing functionalities. In 2020, Feng et al. developed a hybrid nanogenerator that combined a soft contact cylindrical TENG with an oscillating structure EMG for the efficient conversion of ultra-low-frequency wave energy [145]. The incorporation of a flexible rabbit fur brush into separate stator–rotor pairs served the purpose of inducing a charge transfer to the dielectric surface, thereby reducing operational resistance and enhancing device durability. The year 2022 witnessed the unveiling of a hybrid triboelectric–electromagnetic nanogenerator (FH-HG) by Han et al., featuring a double-sided villus and dual Halbach array structure for the efficient harvesting of ultra-low-frequency wave energy [146]. The incorporation of the double-sided pile and dual Halbach array design ensured exceptional performance at low frequencies, while simultaneously extending its battery life. In 2022, Zhang et al. integrated the lightweight TENG with the heavy PENG and EMG, thereby enhancing both the water wave energy harvesting capability of the hybrid device and optimizing module space utilization [148]. Moreover, this module effectively captured both kinetic and gravitational potential energy from water waves by leveraging the dual oscillating degrees of freedom offered by a two-line pendulum. In 2024, Zhai et al. developed a highly sensitive modular spindle hybrid nanogenerator (MSHG) comprising contact-separated triboelectric nanogenerator (TENG), piezoelectric nanogenerator (PENG), and electromagnetic generator (EMG) components [149]. The integrated unit demonstrated excellent responsiveness to low intensity excitation and exhibited robust energy harvesting capabilities in marine environments. The data in Table 2 demonstrates that hybrid nanogenerators have achieved output power densities of hundreds of watts per cubic meter, indicating significant potential for application in the field of blue energy collection.

## 8. The Challenges of the Different Energy Harvesting Technologies

**Figure 15:**
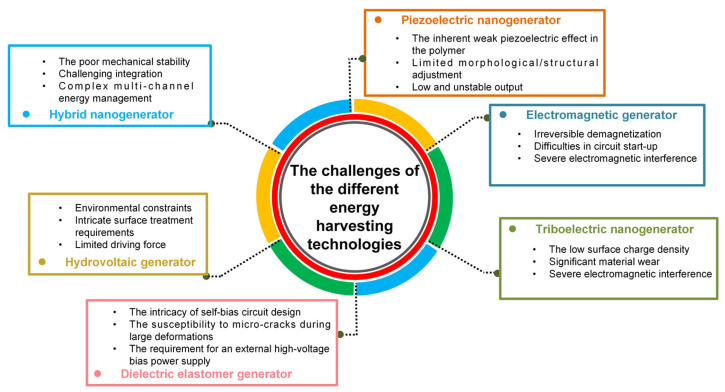
The challenges of the different energy harvesting technologies.

The field of wave energy generation has witnessed rapid development in various conversion mechanisms for energy harvesting devices in recent years; however, their further application still encounters a series of challenges. The challenges encountered by the different energy conversion mechanisms, as depicted in the Figure 15, are summarized in this section.

The three bottlenecks of piezoelectric nanogenerators include the inherent weak piezoelectric effect in the polymer, limited morphological/structural adjustment within a narrow length scale, and a low piezoelectric output with an unstable signal.

(1) **The inherent weak piezoelectric effect in the polymer:** The low piezoelectric output of the prepared PENG (piezoelectric nanogenerator) hinders its extensive application in the Internet of Things, primarily due to inherent properties of the polymer itself.
(2) **Limited morphological/structural adjustment:** The limited adjustment of the shape and structure of the material within a specific size range restricts the enhancement of piezoelectric properties, thus falling short of meeting the required performance standards.
(3) **Low and unstable output:** The polymer’s inherent weak piezoelectric effect leads to signals that lack stability and strength, rendering them inadequate for certain applications.

The development of electromagnetic nanogenerators faces three major challenges: irreversible demagnetization, difficulties in circuit start-up, and severe electromagnetic interference.

(1) **Irreversible demagnetization:** The improper design and utilization of an electromagnetic nanogenerator can lead to irreversible demagnetization, resulting in the loss of magnetism due to excessively high or low temperatures, an armature reaction caused by the shock current, or intense mechanical vibrations. Consequently, this may result in device degradation or render it non-functional.
(2) **Difficulties in circuit start-up:** The output voltage of the electromagnetic nanogenerator is significantly low, even at the mV level, under weak excitation. Consequently, this hinders the normal start-up of traditional tube management modules.
(3) **Severe electromagnetic interference:** When constructing a self-powered system, the presence of a permanent magnet in the electromagnetic nanogenerator may result in electromagnetic interference to the signal of the sensor as well as the signal acquisition and processing unit.

The challenges in the development of triboelectric nanogenerators include the low surface charge density, significant material wear, and difficulties in measuring high impedance signals.

(1) **The low surface charge density:** Although the triboelectric nanogenerator possesses the advantages of a cost-effective preparation and seamless integration, it has encountered a bottleneck issue in terms of low surface charge density, which hampers its widespread application and adoption.
(2) **Significant material wear:** Material wear and heating at the friction interface will affect the durability of the device, especially for rotary and sliding triboelectric nanogenerators.
(3) **Difficulties in measuring high impedance signals:** The electrical output of the TENG is characterized by a high voltage, high impedance, and low current, posing challenges in terms of energy extraction and information acquisition.

The main challenges in the development of dielectric elastomer generators include the requirement for an external high-voltage bias power supply, the susceptibility to micro-cracks during large deformations, and the intricacy of self-bias circuit design.

(1) **The requirement for an external high-voltage bias power supply:** The output performance of the conventional dielectric elastomer generator is contingent upon the external high-voltage power supply and its substantial shape variability, thereby constraining its applicability and augmenting system intricacy and cost.
(2) **The susceptibility to micro-cracks during large deformations:** The dielectric elastomer film is prone to generating micro-cracks within its structure when subjected to significant deformation, thereby compromising its longevity and performance while constraining its ability to sustain continuous operation under such conditions.
(3) **The intricacy of self-bias circuit design:** To address the issue of an external high-voltage bias power supply, researchers have explored the utilization of self-bias; however, this approach necessitates an intricate circuit design and multiple mechanical cycles for achieving a high-voltage DC output, thereby augmenting the system complexity and energy losses.

The main challenges of the hydrovoltaic generator lie in its limited driving force, intricate surface treatment requirements, and environmental constraints.

(1) **Limited driving force:** The slow evaporation rate of water molecules in the environment (small driving force) is the primary factor that limits the efficiency of electricity generation in water volt devices.
(2) **Intricate surface treatment requirements:** The complexity of nanostructure design or surface functionalization represents one of the key technologies for enhancing electricity generation performance.
(3) **Environmental constraints:** Geographical environmental constraints are among the factors that impact the long-term and sustainable production capacity realization of hydrovoltaic generators.

The poor mechanical stability, challenging integration, and complex multi-channel energy management pose bottlenecks for hybrid nanogenerators.

(1) **The poor mechanical stability**: The mechanical stability of hybrid nanogenerators is crucial for ensuring consistent performance over extended periods of use. However, due to the unique properties inherent in nanomaterials, maintaining this stability can be challenging, particularly when subjected to repeated deformation or prolonged operation. Consequently, there may be a decline in performance or even failure.
(2) **Challenging integration**: The integration of various power generation units into a single system necessitates precise control and intricate technology. This entails not only the selection and optimization of materials but also encompasses multiple aspects such as structural design and manufacturing processes. The high level of integration difficulty translates to increased costs and reduced throughput, posing a significant challenge for large-scale applications.
(3) **Complex multi-channel energy management**: The electrical output performance of different energy collectors varies significantly. The realization of the cooperative collection of multi-channel energy and the management of interference between energy sources pose bottlenecks.

## 9. Summary and Outlook

In recent years, there has been a significant surge in the development of micro-energy technology-based wave energy harvesting, indicating its growing prominence in the field. The present paper provided a comprehensive description of the detailed application of several novel micro-energy technologies in wave energy harvesting. The advantages and disadvantages of each form are presented in Table 3. The piezoelectric nanogenerator possesses the advantages of a simplistic structure, facile miniaturization, and convenient array integration; however, its output performance is suboptimal when subjected to low-frequency wave energy excitation, necessitating the incorporation of a coupled extension frequency structure. The electromagnetic generator exhibits high output power; however, it is characterized by a large and bulky volume, as well as poor low-frequency output characteristics. The triboelectric nanogenerator boasts several advantages, including low cost, wide availability of materials, and a broad frequency range for operation. However, its output is susceptible to environmental factors and the reliability of its materials remains a concern in the field of blue energy. The dielectric elastomer generator exhibits the advantages of lightweight, simple structure, and high energy density; however, it necessitates an external power supply for operation and its output performance is constrained by the dielectric breakdown of the material. The hydrovoltaic generator exhibits the dual capability of seawater desalination and electricity generation, featuring a simplistic design and lightweight construction. However, it is accompanied by high costs and susceptibility to pollution from marine environments. The hybrid nanogenerator exhibits a high power density by harnessing the combined advantages of diverse nanogenerators. However, the current development faces a bottleneck issue in effectively managing multiple energy sources. Future development prospects of wave energy harvesting technology include the following: (1) The structural design is progressively evolving towards miniaturization and customization, aiming to cater to the on-site power supply demands of offshore equipment. (2) From the perspective of conversion mechanisms, the mechanism is gradually evolving towards diversification, compounding, and coupling, thereby enhancing wave energy power generation. The emergence of this phenomenon is expected to stimulate a surge in research on the management of multi-channel energy collaboration.

## Figures and Tables

**Table 1:** Several typical instances of dielectric elastic generators employed for the purpose of wave energy harvesting.

References	Materials	Dimensions(cm^3^)	Power Density
[109]	acrylic polymer	ϕ30 × 20	745.91 J/m^3^
[110]	70,410, tesa	\	0.14 J/g
[111]	silicone rubber	ϕ6 × 3	7.322 J/g
[112]	acrylic polymer	ϕ39 × 19	0.3–0.5 J/g
[19]	polyacrylate film	4.63	0.145 J/g
[108]	acrylic elastomer	ϕ60 × 160	2099.96 w/m^3^

**Table 2:** The development of hybrid nanogenerators in recent years.

Author	Type	Year	Power Density	References
L. He, et.al	PENG-EMG	2024	6.9 W/m^3^	[142]
L. Lu, et.al	PENG-EMG	2023	47.83 W/m^2^	[143]
J. Li, et.al	TENG-PENG	2023	7.634 W/m^3^	[144]
Y. Feng, et.al	TENG-EMG	2021	10.16 W/m^3^	[145]
C. Han, et.al	TENG-EMG	2022	18.98 W/m^3^	[146]
S. Ding, et.al	TENG-EMG	2024	37.105 W/m^3^	[147]
C. Zhang, et.al	EMG-PENG-TENG	2022	385.5 W/m^3^	[148]
L. Zhai, et.al	EMG-PENG-TENG	2024	48.2 W/m^3^	[149]
B. Yang, et.al	EMG-PENG-TENG	2024	25.99 W/m^3^	[150]

**Table 3:** Comparative analysis of the advantages and disadvantages associated with various types of energy collectors.

Type	Strengths	Weaknesses
Piezoelectric nanogenerator	Simple structure	Low output power
	Easy miniaturization	Poor low-frequency characteristics
	Easy array	Poor durability
Electromagnetic generator	High conversion efficiency	Large volume
	Not affected by the environment	Bulky Poor low-frequency characteristics
Triboelectric nanogenerator	Simple structure, wide selection of materials	Poor durability
	High conversion efficiency	Vulnerable to environmental impact
	Wide operating frequency band	Impedance matching difficulty
Dielectric elastomer generator	Light weight	Need external power supply
	Simple structure	Easy to breakdown
	High energy density	
Hydrovoltaic generator	Simple structure	Low output power
	Light weight	Susceptible to environmental influences
	Easy to array	
Hybrid nanogenerator	Highly integrated	Multi-channel energy management is difficult
	Synthesize the advantages of various forms	
	High output power	

## Data Availability

Data sharing not applicable.
